# Dynamics of double bubbles under the driving of burst ultrasound

**DOI:** 10.1016/j.ultsonch.2022.105952

**Published:** 2022-02-14

**Authors:** Xun Wang, Weizhong Chen, Min Zhou, Zekun Zhang, Lingling Zhang

**Affiliations:** a Kaiserslautern Intelligent Manufacturing School, Shanghai Dianji University, Shanghai 201306, China; b Key Laboratory of Modern Acoustics, Ministry of Education, Institute of Acoustics, Nanjing University, Nanjing 210093, China; c School of Science, Xi’an Polytechnic University, Xi’an 710048, China

**Keywords:** Burst ultrasound, Double bubble dynamics, Ambient radius, Bubble translation

## Abstract

This paper investigates the pulsations and translation of bubbles in a double-bubble system driven by burst ultrasound. Results illustrate that for two identical bubbles, decreasing the frequency of burst or increasing its amplitude can enhance the pulsations and improve the translation velocities of bubbles. In a certain scope, large bubble brings about fast translation velocity, but the velocity will fall down for too large bubble, such as the bubble with ambient radius over about its resonance radius. When the ambient radii of two bubbles are different, translation of the large bubble is smaller than that of the small bubble. In addition, the effect of initial distance between bubbles is described as well. If burst serials are used, shortening the time interval between each burst and improving the acoustic amplitude of bursts are beneficial for the translations of bubbles.

## Highlights

• Dynamics of double bubbles driven by burst ultrasound rather than continuous wave are discussed.
• Improving the frequency and amplitude of driving brings about fast translation speeds for bubbles.
• The relation between ambient radii of bubbles and the resonance radius has significant effect on the approaching speeds of bubbles.
• If burst serials are used, shortening the time interval between each burst and improving the acoustic amplitude of bursts are beneficial for the translation of bubbles.

## 1 Introduction

The gas nuclei in liquids will grow to be visible bubbles under the driving of ultrasound with enough acoustic pressure. This phenomenon is called ultrasonic cavitation [1], [2], [3]. Pulsation of bubbles will generate high temperature and pressure therein. When the bubbles collapse, shock waves will be produced [4], [5]. That makes cavitation being widely used in all kinds of fields, such as therapy [6], catalyzing [7], wastewater treatment [8] and parts cleaning [9]. Moreover, bubbles can be used in material processing in the reduced gravity environment [10] and drugs delivery for tissues [11] if they can be manipulated. Exploring the dynamics of bubbles is of great significance for the application of them.

In practical application, there usually exists large number of bubbles in the liquid [12]. Unfortunately, understanding and manipulating bubble group are uneasy. Double-bubble system is the simplest form of multi-bubble system. Investigating the double-bubble dynamics is beneficial for exploring the dynamics of bubble group. Some publications suggest that burst ultrasound can improve chemical reaction efficiency [13], [14]. Besides, burst ultrasound is widely used with microbubbles in medical treatment [15]. As a result, it is necessary to investigate the dynamics of bubbles driven by burst ultrasound.

During these years, many researchers have explored the dynamics of double bubbles. Ida [16] discussed the pulse waves radiated by the bubbles in double-bubble system. They found the positive pulse generated by the collapsing of one bubble hit and compressed another bubble to generate reflected negative pulses. Sadighi-Bonabi et al. [17] numerically studied the pulsations of double bubbles in sulfuric acid solution and discussed the secondary Bjerknes force between the two bubbles. They found the direction and magnitude of secondary Bjerknes force are closely related to the viscosity of liquids. Moreover, they concluded the secondary Bjerknes force will increase with viscosity. Zhang et al. [18] numerically investigated the secondary Bjerknes force between two bubbles driven by dual-frequency ultrasound, and discussed the influences of ambient radii, amplitude of driving ultrasound pressure and the phase difference between the two driving. Besides, they compared the results to the data simulated under one driving ultrasound. Shen et al. [19] discussed the suppression or enlargement property of expansion ratios of bubbles in double-bubble system under the driving of ultrasound. They found the interaction between bubbles can not only decrease or suppress the expansion ratios of bubbles, but also enlarge that of bubbles. Besides, they derived the model to describe pulsations of bubbles in liquid confined in the elastic sealed vessel and studied the dynamics of two bubbles [20]. Liang et al. [21] investigated the dynamics of two nonspherical bubbles in the asymmetric ultrasound field. Bubble pulsation and secondary Bjerknes force are the focus of discussion in this paper.

All of the investigations mentioned above are based on the assumption that the positions of bubbles are fixed. In real situation, the bubbles will move due to the secondary Bjerknes force between bubbles [22]. Some researchers have explored the dynamics of double-bubble system considering the moving of bubbles. Doinikov [23] deduced the equations of radial and translational motions of two coupled bubbles with Lagrangian formalism. The simulation results show if the driving is strong enough, the two bubbles will get close and move with a steady spacing rather than collide and coalesce. Zhang et al. [24] introduced the bifurcations of radii and positions of double bubbles based on Doinikov’s model. Bremond et al. [25] investigated the combination process of two bubbles with high speed camera, and compared the bubble pictures recorded during experiments to simulation results. Cai et al. [26] studied the translations and pulsations of two encapsulated bubbles driven by ultrasound with finite element simulation method, and discussed the bubble dynamics in details. Results show the translations are closely related to the resonant frequencies of bubbles and the frequency of ultrasound. If the ultrasound frequency locates between the resonant frequencies of the two bubbles, the two bubbles will attempt to repel each other. Otherwise the two bubbles are attractive to each other. Recently, Qin et al. [27] revised Doinikov’s model to exploring the nonlinear dynamics and acoustic emissions of double-bubble system in viscoelastic tissues. Bifurcations of radii of bubbles under different parameters have been heavily discussed. Liang et al. [28] proposed the equations which can describe the pulsation and moving of nonspherical bubbles with perturbation theory. Numerical simulations show the pulsations, translations and deformations of the two bubbles are periodic. The pulsation periods of two bubbles decrease with the increasing of initial translation velocities of bubbles. Zhang et al. [29] deduced the bubble dynamics equations when the heterogeneity of pressure exerting on the bubbles are considered. Simulations show the two bubbles will get close if the difference of their pulsing phases is small, and will repel to each other if their pulsing phases are opposite.

To sum up, although there exists many publications describing the dynamics of double-bubble system, driving in these publications are continuous acoustic wave. For the burst ultrasound, each burst only exists for a short duration. The dynamics of double bubbles driven by burst ultrasound have hardly been explored so far. In the present study, the pulsations and moving of two bubbles in double-bubble system are discussed. The remainder of this article is organized as follows: In Section 2, the equations which describe the bubble dynamics are introduced. In Section 3, the effects of burst ultrasound with different parameters on bubble dynamics are introduced. In Section 4, the pulsations and translations of bubbles with different ambient radii and initial conditions are discussed. In Section 5, dynamics of double bubbles driven by burst serials are explored. Conclusions are summarized in Section 6.

## 2 Mathematical Model

If the translations of bubbles in double-bubble system are considered, dynamics of two bubbles can be described by Doinikov Model [23]

(1) $$\left(1-\frac{{\dot{R}}_{i}}{c}\right)R_{i}{\ddot{R}}_{i}+\left(\frac{3}{2}-\frac{{\dot{R}}_{i}}{2c}\right){\dot{R}}_{i}^{2}-\frac{1}{\rho }\left(1+\frac{{\dot{R}}_{i}}{c}\right)p_{i}-\frac{R_{i}}{\rho c}\frac{dp_{i}}{dt}=\frac{{\dot{x}}_{i}^{2}}{4}-\frac{R_{3-i}^{2}{\ddot{R}}_{3-i}+2R_{3-i}{\dot{R}}_{3-i}^{2}}{D}+{\left(-1\right)}^{3-i}\frac{R_{3-i}^{2}\left({\dot{x}}_{i}{\dot{R}}_{3-i}+R_{3-i}{\ddot{x}}_{3-i}+5{\dot{R}}_{3-i}{\dot{x}}_{3-i}\right)}{2D^{2}}-\frac{R_{3-i}^{3}{\dot{x}}_{3-i}\left({\dot{x}}_{i}+2{\dot{x}}_{3-i}\right)}{2D^{3}},$$

(2) $$\frac{R_{i}{\overset{¨}{x}}_{i}}{3}+{\overset{˙}{R}}_{i}{\overset{˙}{x}}_{i}+{\left(-1\right)}^{3-i}\frac{1}{D^{2}}\frac{d}{dt}\left(R_{i}R_{3-i}^{2}{\overset{˙}{R}}_{3-i}\right)-\frac{R_{3-i}^{2}\left(R_{i}R_{3-i}{\overset{¨}{x}}_{3-i}+R_{3-i}{\overset{˙}{R}}_{i}{\overset{˙}{x}}_{3-i}+5R_{i}{\overset{˙}{R}}_{3-i}{\overset{˙}{x}}_{3-i}\right)}{D^{3}}=\frac{F_{\mathrm{ex}i}}{2\pi \rho R_{i}^{2}},i=1,2,$$

with $R_{i}$ and $x_{i}$ being the radius and position of the *i*th bubble, respectively, *D* being the distance between the two bubbles, ρ being the density of the liquid, *c* being the sound speed in the liquid. $F_{\mathrm{ex}i}$ is the force driving the *i*th bubble to move. Eq. (1) and Eq. (2) decide the pulsations and translations of the bubbles, respectively. In Eq. (1), $p_{i}$ satisfies

(3) $$p_{i}=\left(P_{0}+\frac{2\sigma }{R_{i0}}\right){\left(\frac{R_{i0}}{R_{i}}\right)}^{3\gamma }-\frac{2\sigma }{R_{i}}-\frac{4\eta {\dot{R}}_{i}}{R_{i}}-P_{0}-P_{t},$$

with $P_{0}$ being the ambient pressure, $R_{i0}$ being the ambient radius of the *i*th bubble, $P_{t}$ being the driving acoustic pressure, σ being the surface tension coefficient of the liquid, η being the viscosity coefficient of the liquid. $F_{\mathrm{ex}i}$ in Eq. (2) can be expressed as [23], [24]

(4) $$F_{\mathrm{ex}i}=-12\pi \eta R_{i}\left({\dot{x}}_{i}+\frac{{\left(-1\right)}^{3-i}R_{3-i}^{2}{\dot{R}}_{3-i}}{D^{2}}-\frac{R_{3-i}^{3}{\dot{x}}_{3-i}}{D^{3}}\right)\text{.}$$

In this paper, the driving $P_{t}$ is the acoustic pressure of burst ultrasound which can be expressed as

(5) $$P_{t}=P_{d}\cos\left(2\pi \mathrm{ft}\right)\left[\frac{1}{2}-\frac{1}{2}\cos\left(2\pi f\frac{t}{N}\right)\right],$$

with $P_{d}$ and *f* being the amplitude and the center frequency of burst, respectively. *N* decides the number of acoustic cycles in the burst. The secondary Bjerknes force exerted by the $\left(3-i\right)$th bubble to the *i*th bubble is given by [17], [24]

(6) $$F_{\left(3-i\right)i}=\frac{\rho }{4\pi D^{2}}V_{i}\frac{d^{2}V_{3-i}}{dt^{2}},$$

where $V_{i}$ is the volume of the *i*th bubble. The radii and positions of two bubbles can be acquired by solving Eqs. (1), (2), (3), (4), (5) with Runge–Kutta method.

## 3 Effect of single burst wave

### 3.1 Effect of frequency

**Fig. 1:**
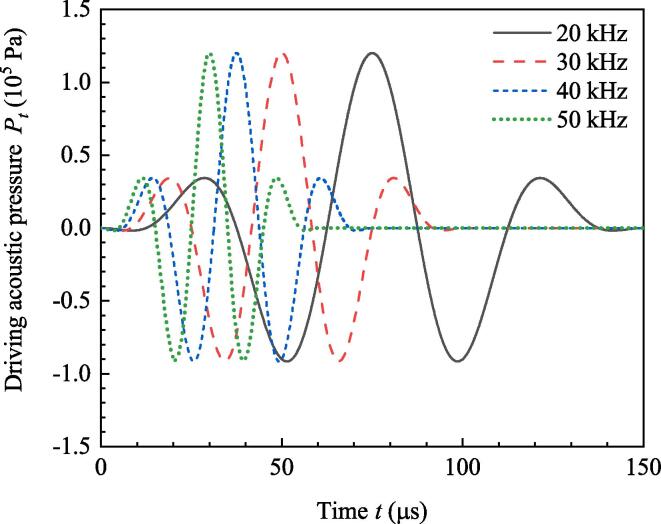
Acoustic pressures of ultrasound burst with different frequencies.

Fig. 1 illustrates the acoustic pressure when *f* are 20 kHz, 30 kHz, 40 kHz and 50 kHz, resepctively. It can be found that the duration time of a burst when f=20kHz is the longest and that when f=50 kHz is the shortest. What can be easily inferred is that if *N* in Eq. (5) is fixed, high *f* makes the duration time of burst short. Moreover, frequency has no influence on the amplitude of burst.

**Fig. 2:**
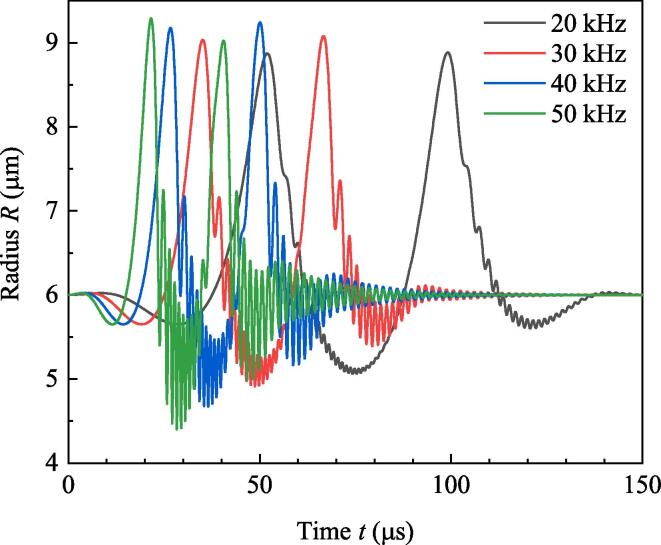
Radii of bubbles driven by burst ultrasound of different frequencies.

**Fig. 3:**
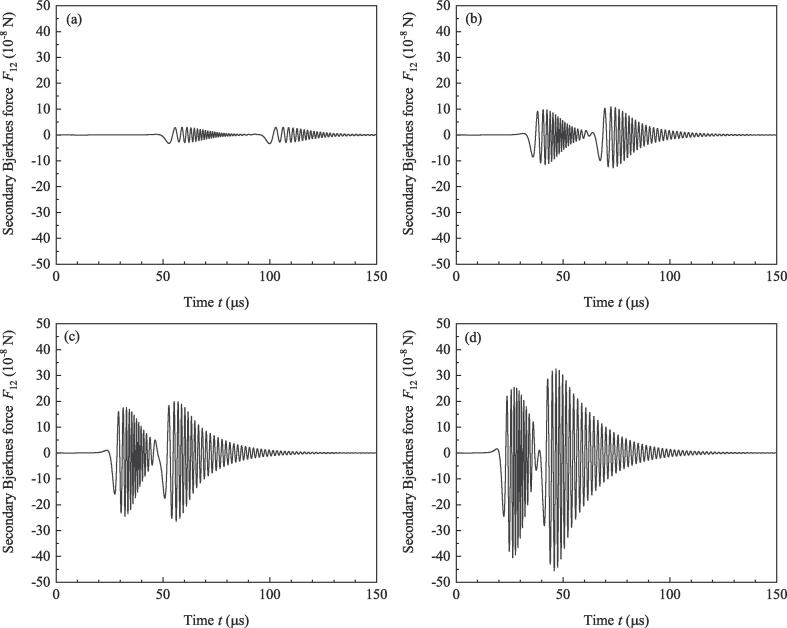
Secondary Bjerknes force exerted from bubble 1 on bubble 2 (the same as that from bubble 2 on bubble 1). (a) f=20kHz, (b) f=30kHz, (c) f=40kHz, (d) f=50kHz.

**Fig. 4:**
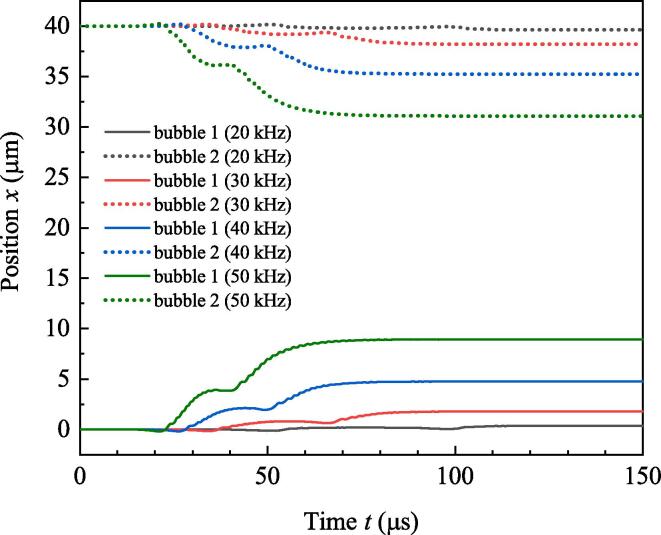
Positions of bubbles driven by burst ultrasound of different frequencies.

Simulation is done for numerically investigating the effect of ultrasound burst frequency. Parameters used in simulation are as follows: $R_{10}=R_{20}=6\mu m,\rho =998\mathrm{kg}/m^{3},c=1500m/s,P_{0}=1.013\times 10^{5}$ Pa, $P_{d}=1.2\times 10^{5}$ Pa, σ=0.0725N/m,η=0.001Pa·s,γ=1.4,N=3. The initial values of $x_{1}$ and $x_{2}$ are 0 and 40μm, respectively, so the initial value of *D* ($D_{0}$) is 40μm. Set the total simulation time to be 150μs and do simulation under the driving of the above-mentioned four frequencies. From the vanishing moment of burst to the end of simulation, $P_{t}$ keeps to be zero. Because the initial conditions of the two bubbles are the same, radii of the two bubbles are overlapped, and the amplitudes of secondary Bjerknes force satisfy $F_{12}=F_{21}$. Fig. 2 shows the variations of radii. It can be found that high frequency brings about strong pulsations for bubbles. According to the resonance frequency formula $f_{0}=\frac{1}{2\pi R_{0}}\sqrt{\frac{3\gamma P_{0}}{\rho }+\frac{2\left(3\gamma -1\right)\sigma }{\rho R_{0}}}$ in Ref.[30], resonance frequency of bubble with ambient radius 6μm is about 595 kHz. When *f* approaches to resonance frequency, resonance oscillation tends to be happen for bubbles. So in Fig.2, high driving frequency means strong pulsations of bubbles. But if *f* is larger than the resonance frequency, high driving frequency generates weak pulsations of bubbles (not shown). Fig. 3 shows the secondary Bjerknes force exerted from bubble 1 on bubble 2 (the same as that from bubble 2 on bubble 1). With the increasing of *f*, amplitude of secondary Bjerknes force becomes large. In double-bubble system, if the two bubbles are the same, they will be attractive to each other [29]. So large secondary Bjerknes force means strong attraction. Fig. 4 shows the positions of bubbles under the driving of different frequencies. It can be found that high frequency driving makes the bubbles approach fast, so the final distance between bubbles under high frequency driving is smaller than that under low frequency driving at the end of simulation.

### 3.2 Effect of acoustic amplitude

**Fig. 5:**
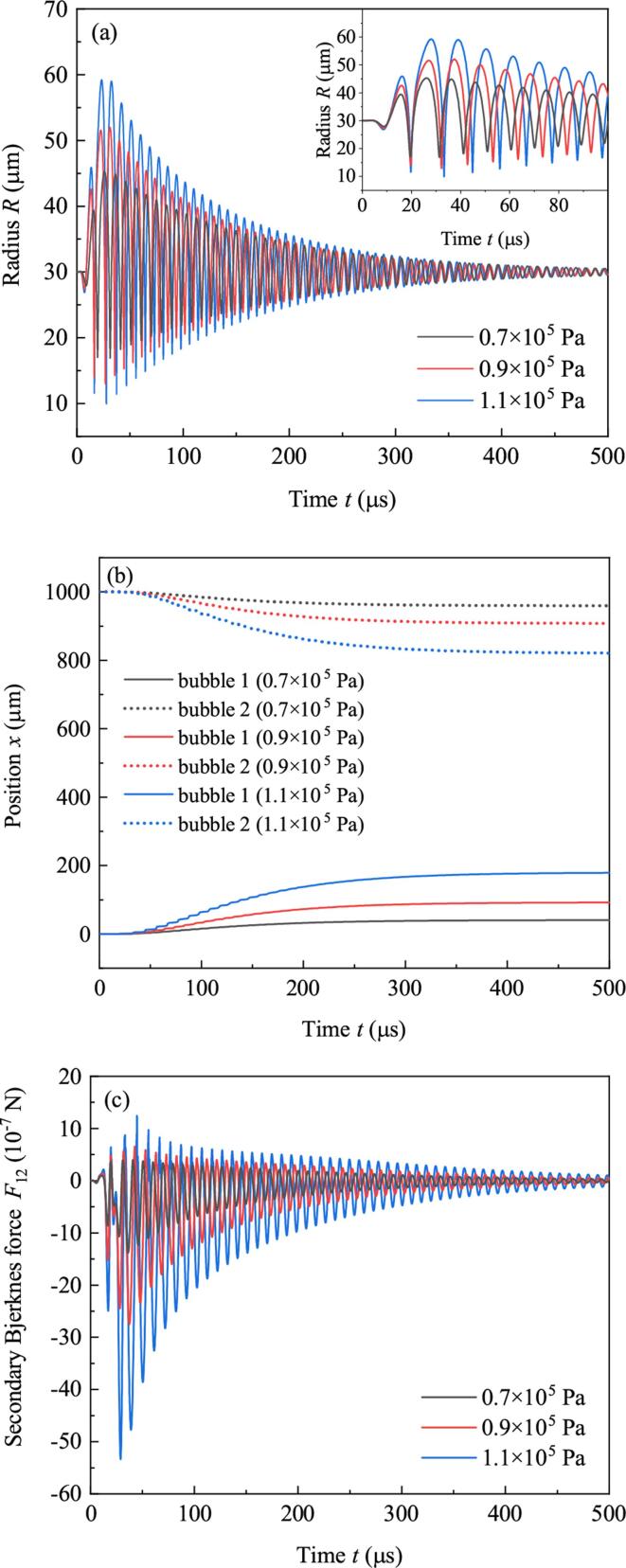
Bubble dynamic characteristics under driving of different amplitudes. (a) Radii, (b) positions, (c) secondary Bjerknes force.

Set f=80 kHz and $R_{10}=R_{20}=30\mu$m, and set the amplitude of driving ($P_{d}$) to be $0.7\times 10^{5}$ Pa, $0.9\times 10^{5}$ Pa and $1.1\times 10^{5}$ Pa, respectively. Fig. 5 shows the calculated radii (Fig.5(a)), positions (Fig.5(b)) and the secondary Bjerknes force (Fig.5(c)) of bubbles when the initial distance between bubbles ($D_{0}$) is 1000μm. From Fig.5(a), it can be found that with the increasing of $P_{d}$, the minimum radii when bubbles collapse decrease, and the maximum radii in the expansion phases of bubbles increase in each pulsation period. That means large $P_{d}$ produces strong pulsations of bubbles. From Fig.5(b), it can be concluded that high $P_{d}$ corresponds to fast approaching speeds and large displacements of bubbles. In Fig.5(c), the amplitude of $F_{12}$ (the same as $F_{21}$) increases with $P_{d}$. So the fast approaching speeds can be attributed to the strong secondary Bjerknes force produced by drastic pulsations of bubbles.

### 3.3 Effect of cycle number in a burst

**Fig. 6:**
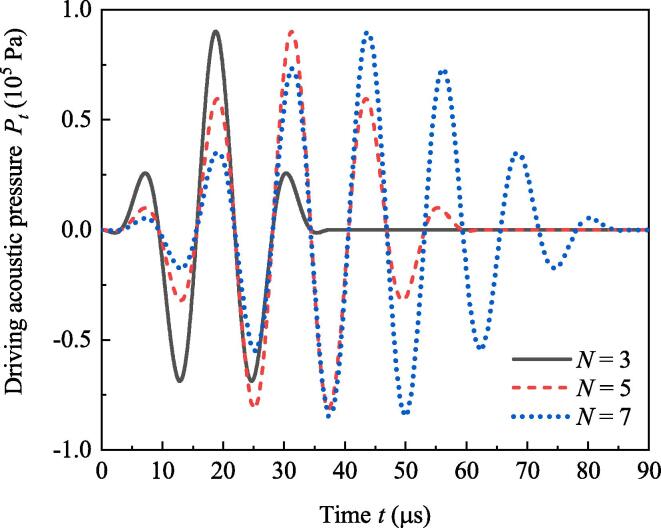
Acoustic pressures of burst waves with different acoustic cycles.

**Fig. 7:**
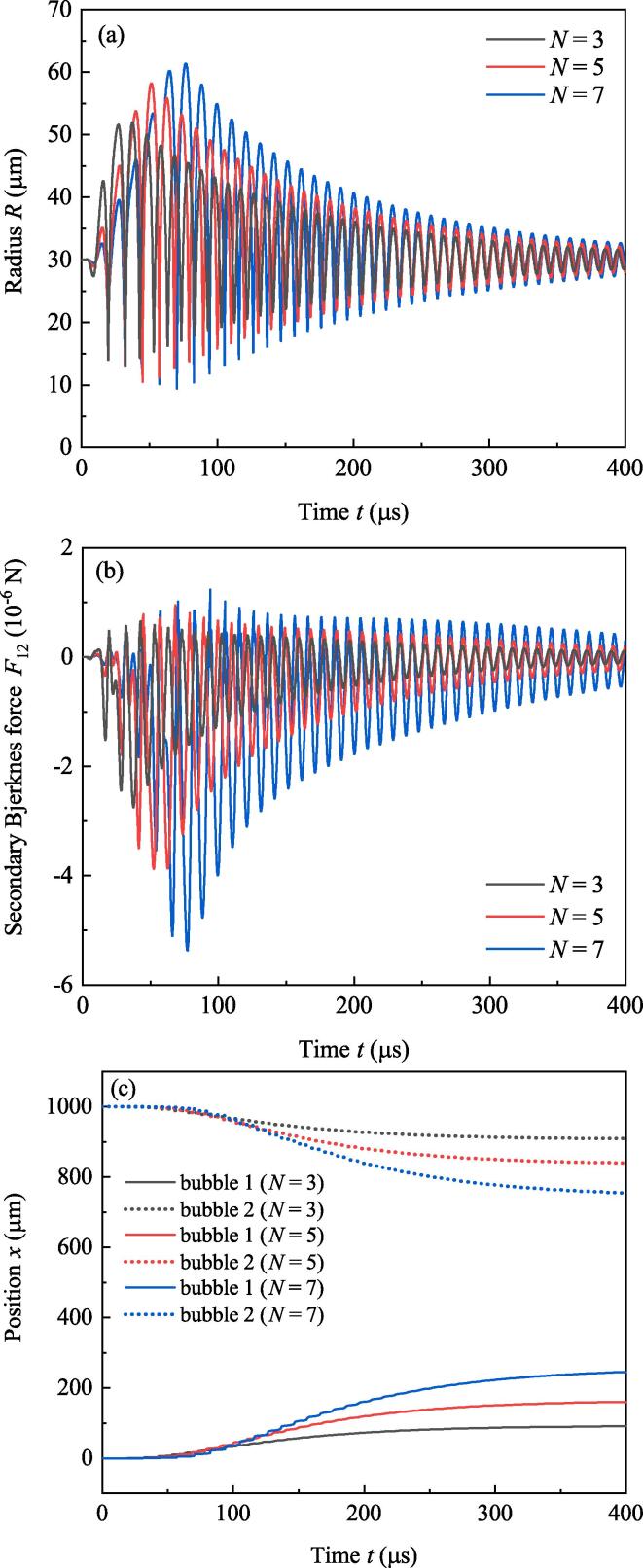
Radii, positions and secondary Bjerknes force of bubbles under the driving of burst waves last for different acoustic cycles. (a) Radii, (b) secondary Bjerknes force, (c) positions.

Fig. 6 exhibits the acoustic pressures of burst waves when $f=80\mathrm{kHz},R_{10}=R_{20}=30\mu m$ and the number of cycles (*N*) in Eq. (5) is 3, 5 and 7, respectively. It is obvious that large *N* corresponds to burst with long duration. Fig. 7 shows the radii (Fig.7(a)), secondary Bjerknes force (Fig.7(b)) and positions (Fig.7(c)) of bubbles. If *N* becomes large, maximum bubble radii at the initial stage (t<40μs) decrease. But when t>70μs, large *N* corresponds to large maximum radii and small minimum radii of bubbles (see Fig. 7(a)). That means in majority proportion of simulation time, burst wave with large *N* results in strong pulsations of bubbles. In Fig.7(b), large *N* brings about large secondary Bjerknes force when t>70μs. That is to say large *N* creates significant attraction for bubbles. As a result, when t>70μs, approaching speeds of bubbles driven by burst wave with large *N* are faster than that with small *N*, as well as the displacements of two bubbles (see Fig. 7(c)).

## 4 Effects of ambient radii of bubbles and initial distance between bubbles

### 4.1 Effect of ambient radii of bubbles

**Fig. 8:**
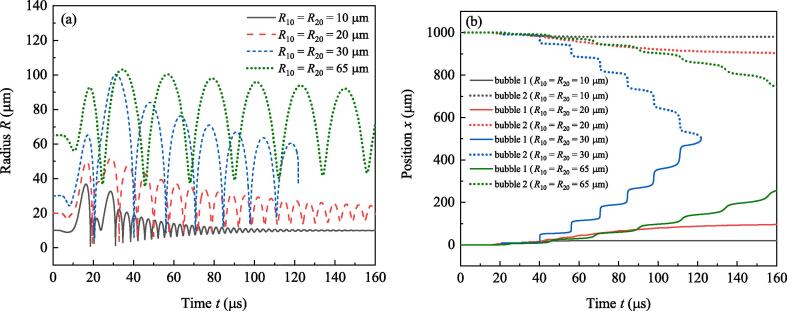
Radii and positions of bubbles when the ambient radius of bubble 1 takes different values. (a) Radii, (b) Positions.

In this section, discuss focuses on the impact of bubbles’ ambient radii. When f=80 kHz and $R_{10}=R_{20}$, suppose the ambient radii of the two bubbles to be 10μm,20μm,30μm and 65μm, respectively, and do the simulation. Fig.8 illustrates the calculated radii (Fig.8(a)) and positions (Fig.8(b)) of bubbles. In Fig.8(a), when $R_{10}$ and $R_{20}$ vary from 10μm to 30μm , the maximum radii of bubbles in each pulsation cycle increase obviously, whereas the minimum radii change a little. That means large $R_{10}$ and $R_{20}$ bring about drastic pulsations for bubbles, so the approaching speeds of bubbles increase with ambient radii of bubbles (see Fig.8(b)). However, if the ambient radii exceed 40μm, increasing of them will generate negative effect on approaching speeds. For example, when $R_{10}=R_{20}=65\mu m$, the minimum radii in every cycle become large apparently comparing with that when $R_{10}=R_{20}=30\mu m$, and approaching speeds of bubbles are obviously smaller than that when $R_{10}=R_{20}=30\mu m$ (see Fig.8(b)). According to the linear resonance frequency formula in Ref.[30], the resonance radius corresponds to 80kHz driving is about 42μm. Based on these analysis, it can be inferred that if the ambient radii of bubbles are significantly larger than the resonance radius, pulsations of bubbles become weak and the attraction force between bubbles falls down with the increasing of ambient radii.

**Fig. 9:**
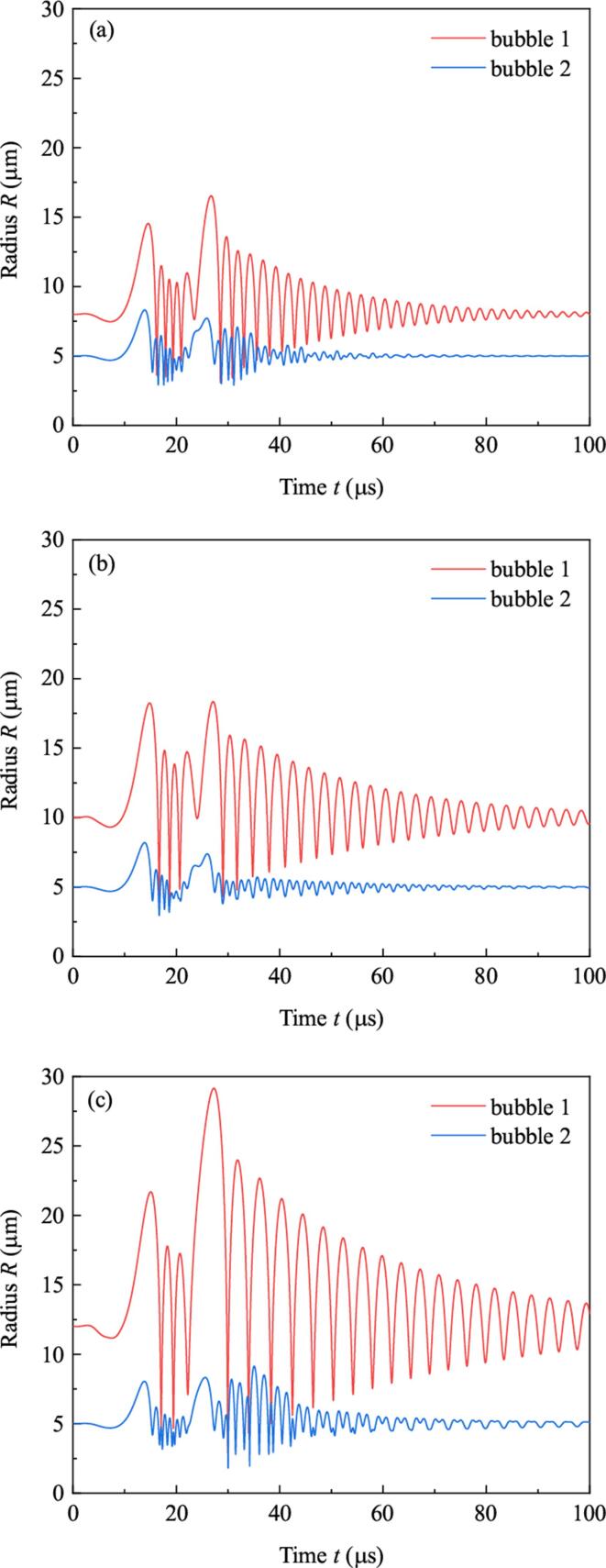
Radii of bubbles when $R_{10}$ takes diverse values. (a)$R_{10}=8\mu m$, (b)$R_{10}=10\mu m$, (c)$R_{10}=12\mu m$.

**Fig. 10:**
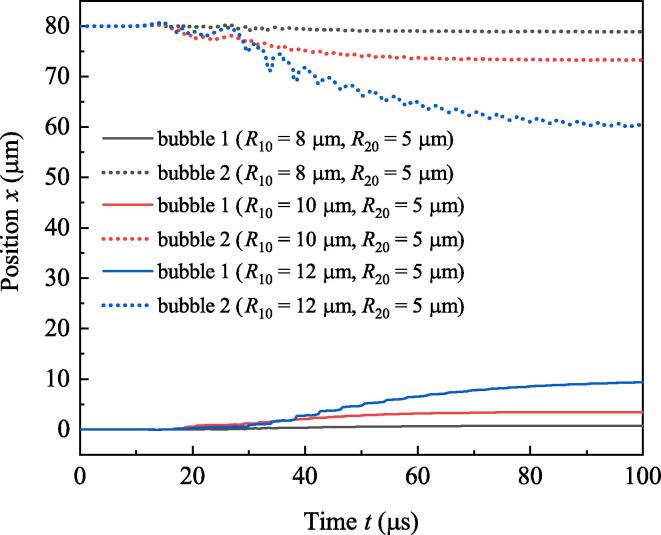
Positions of bubbles when $R_{10}$ takes diverse values.

In the above study, ambient radii of the two bubbles are supposed to be equivalent. If $R_{10}\;\neq \;R_{20}$, the dynamics of bubbles will be quite different. Now set $R_{20}=5\mu m$, and do calculation when $R_{10}$ is 8μm,10μm and 12μm, respectively. Variations of radii and positions of the two bubbles are shown in Fig. 9 and Fig.10, respectively. Fig. 9 shows with the increasing of $R_{10}$, pulsation of bubble 1 becomes strong. In Fig.10, with the increasing of $R_{10}$, displacements of both bubbles increase. Moreover, displacement of bubble 1 (large bubble) is smaller than that of bubble 2 (small bubble). It can be concluded that large $R_{10}$ results in strong attraction for both bubbles, and large bubble is relatively hard to be moved. It should be emphasized that this principle is only valid when $R_{10}$ is less than 15μm. If $R_{10}$ becomes too large, the relation between the movements of bubbles and their ambient radii becomes complicated. The details are not included in this paper.

### 4.2 Effect of initial distance between bubbles

**Fig. 11:**
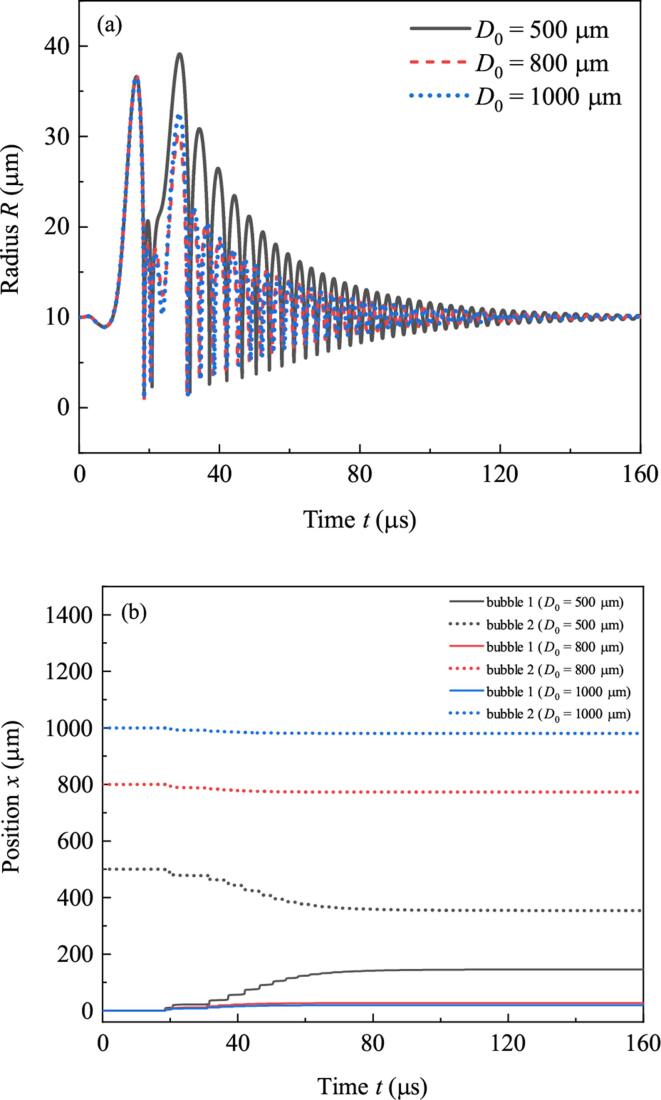
Radii and positions of bubbles when the initial distance between the two bubbles takes different values. (a) Radii, (b) positions.

**Fig. 12:**
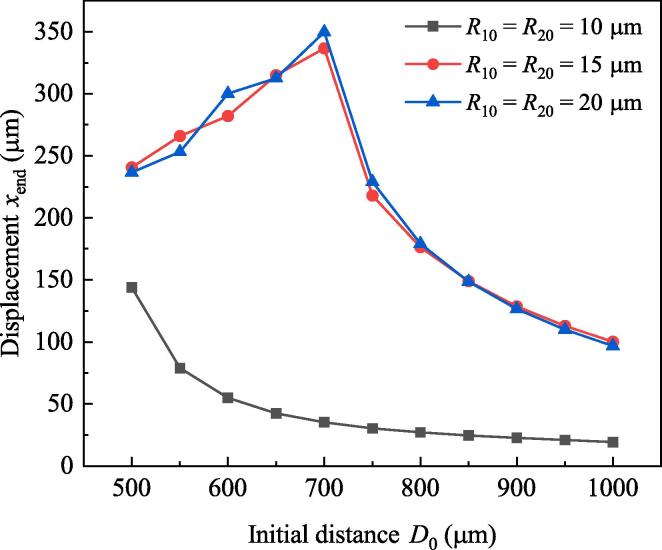
Total translation displacement of each bubble in double-bubble system.

According to the dynamics equations, the distance between bubbles plays important in deciding bubble dynamics. This section focuses on the effect of initial distance $D_{0}$. Suppose $R_{10}=R_{20}=10\mu m,P_{d}=2.3\times 10^{5}\mathrm{Pa}$, setting $D_{0}$ to be 500μm,800μm and 1000μm, respectively and do simulation. Duration of simulation is 160μs. Radii and positions of bubbles are illustrated in Fig.11(a) and Fig.11(b). In Fig.11(a), the maximum radii of bubbles in each cycle when $D_{0}=500\mu m$ are larger than that when $D_{0}=800\mu m$ and $D_{0}=1000\mu m$. Correspondingly, the displacement of each bubble when $D_{0}=500\mu m$ is the largest among the three situations (see Fig.11(b)). Fig. 12 shows the relations between displacements of bubble 1 at the end of simulation ($x_{\mathrm{end}}$) and the initial distances between bubbles when the ambient radii are 10μm,15μm and 20μm, respectively. When $R_{10}=R_{20}=10\mu m,x_{\mathrm{end}}$ decreases with $D_{0}$. It is because large $D_{0}$ means weak bubble pulsation and small secondary Bjerknes force. When $R_{10}=R_{20}=15\mu m$ and $R_{10}=R_{20}=20\mu m,x_{\mathrm{end}}$ grows firstly and then falls down. The reason is that when $D_{0}⩽700\mu m$, the two bubbles will collide at the middle position. So large $D_{0}$ means large translation distance. If $D_{0}>700\mu m$, the bubbles will never meet, large $D_{0}$ means small secondary Bjerknes force and weak attraction between them. As a result, $x_{\mathrm{end}}$ decreases with $D_{0}$.

## 5 Effect of burst serials

In the former discussion, impact of one ultrasound burst has been discussed. In real situation, the bursts are continuous. So in this section, the effect of several burst waves will be intensively introduced.

**Fig. 13:**
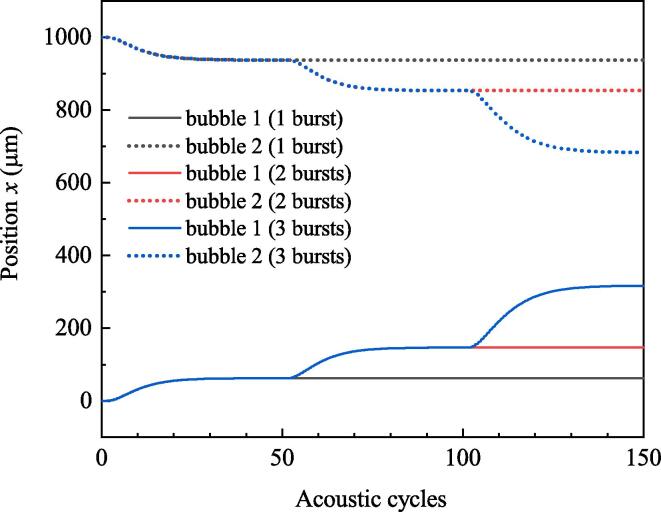
Positions of bubbles when the numbers of burst waves are different.

**Fig. 14:**
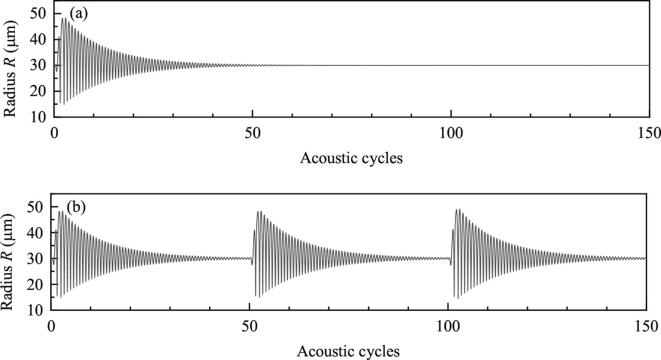
Radii of bubbles when the numbers of burst waves are different. (a) 1 burst wave, (b) 3 burst waves.

Set $f=80\mathrm{kHz},R_{10}=R_{20}=30\mu m,P_{d}=8.0\times 10^{4}\mathrm{Pa}$. Duration of simulation is 150 acoustic cycles (150/f). Time interval from the beginning of a burst to the beginning of the next burst ($T_{N}$) is 50 cycles. Fig. 13 shows the bubble positions when one, two or three bursts are used. If there is only one burst, bubbles will be static after 50 acoustic cycles. They will be moved again under the driving of the subsequent bursts. Moreover, it can be found that the burst wave appears late makes the bubble move for long displacement Fig. 14 shows the radius of one bubble when one or three bursts are used. Variations of bubble radii are almost the same under the driving of each burst wave. Considering the distance between bubbles is small when the late burst wave exerts on, and small distance between bubbles brings about large secondary Bjerknes force, the phenomenon that burst wave appears late makes the translation distances of bubbles large is reasonable.

**Fig. 15:**
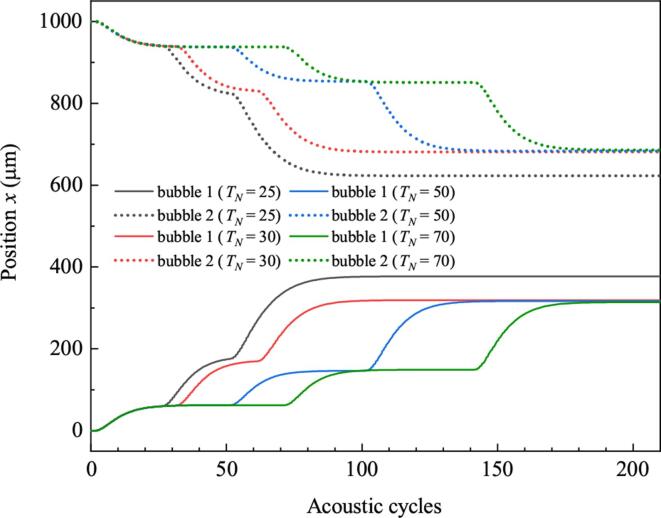
Positions of bubbles when the time interval between each burst wave is different.

Fig. 15 shows positions of bubbles driven by burst serials which composed of three burst waves. $T_{N}$ for the four serials are 25, 30, 50 and 70 cycles, respectively. It should be emphasized that no matter what the interval is, the total energy of the four driving are the same. However, it can be found that displacements of bubbles driven by burst serials with small burst-interval are larger than that with large burst-interval. That means taking use of burst serial with small interval can facilitate the translations of bubbles. It is because for driving with small $T_{N}$, the subsequent burst exerts on bubbles when they are still moving. Kinetic energy comes from the former burst is not completely consumed by liquids. Velocities of bubbles when the burst disappears are fast. But for driving with large $T_{N}$, bubbles are almost static when each burst appears. That means all of the kinetic energy is consumed. Velocities of bubbles when the burst disappears are relatively low. So the total displacements of bubbles driving by burst with large $T_{N}$ are small.

**Fig. 16:**
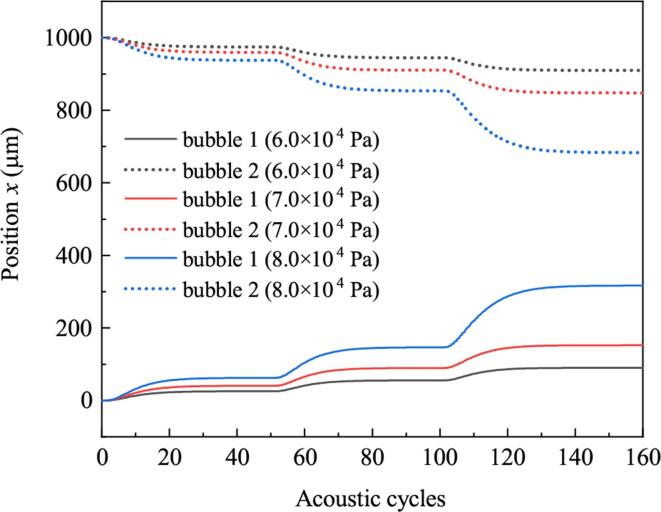
Positions of bubbles when the amplitudes of burst waves are different.

**Fig. 17:**
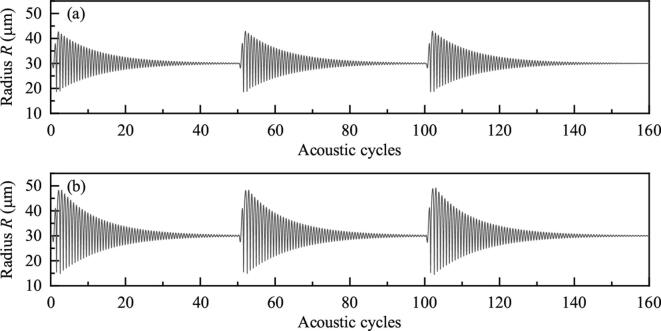
Radii of bubbles when the amplitudes of burst waves are different. (a) $P_{d}=6.0\times 10^{4}\mathrm{Pa}$, (b) $P_{d}=8.0\times 10^{4}\mathrm{Pa}$.

Fig. 16 illustrates the positions of bubbles driven by burst waves of different acoustic amplitudes. With the increasing of amplitude, approaching speeds of bubbles increase. So large amplitude corresponds to small distance between bubbles at the end of simulation. Fig. 17 shows the radii of bubbles when the burst wave amplitudes are $6.0\times 10^{4}\mathrm{Pa}$ and $8.0\times 10^{4}\mathrm{Pa}$, respectively. Radii of bubbles driven by high amplitude burst are obviously larger than that driven by low amplitude burst. So the two bubbles get close fast under high amplitude burst can be attributed to the strong secondary Bjerknes force between them.

## 6 Conclusion

In this paper, the dynamics of double bubbles driven by burst ultrasound are introduced. Effects of the driving are deeply researched and discussed. It has been revealed that high frequency and large amplitude driving burst are beneficial for the translations of bubbles. Increasing the acoustic cycles in one burst can facilitate the translations of bubbles as well. Besides, effects of the ambient radii and initial distance between bubbles are introduced in details. Small ambient radii bring about large displacements of bubbles, and vice versa. Moreover, this paper reveals if the two bubbles do not meet, large initial distance between bubbles means small displacement for each bubble. But if the two bubbles will collide, large initial distance corresponds to large displacement. Investigation on the influence of burst serials shows that shortening the interval between bursts and enlarging the acoustic amplitude of bursts are advantageous for enhancing the translations of bubbles. However, in this research, the shapes of bubbles are supposed to be spherical. In fact, during the pulsation process of bubbles, the force produced by the driving ultrasound may not be uniform. As a result, nonspherical deformations of bubbles could happen. Besides, this research only explores the bubbles in water. In practical application of burst ultrasound, bubbles may exist in the viscous liquid. In the future, the dynamics of nonspherical bubbles will be discussed. Moreover, the dynamics of bubbles in different liquids will be explored.

## CRediT authorship contribution statement

**Xun Wang:** Conceptualization, Methodology, Software, Writing - original draft. **Weizhong Chen:** Methodology, Funding acquisition, Supervision. **Min Zhou:** Writing - review & editing, Funding acquisition. **Zekun Zhang:** Writing - review & editing. **Lingling Zhang:** Writing - review & editing.

## Declaration of Competing Interest

The authors declare that they have no known competing financial interests or personal relationships that could have appeared to influence the work reported in this paper.
